# Twelve tips for successfully implementing logbooks in clinical training

**DOI:** 10.3109/0142159X.2015.1132830

**Published:** 2016-02-03

**Authors:** Katrin Schüttpelz-Brauns, Elisabeth Narciss, Claudia Schneyinck, Klaus Böhme, Peter Brüstle, Ulrike Mau-Holzmann, Maria Lammerding-Koeppel, Udo Obertacke

**Affiliations:** ^a^ Heidelberg University, Germany; ^b^ University of Freiburg, Germany; ^c^ University of Tubingen, Germany

## Abstract

**Background**: Logbooks are widely used to set learning outcomes and to structure and standardize teaching in clinical settings. Experience shows that logbooks are not always optimally employed in clinical training. In this article, we have summarized our own experiences as well as results of studies into twelve tips on how to successfully implement logbooks into clinical settings.

**Methods**: We conducted both a workshop concerning the importance of logbook training to exchange experiences in teaching practice, organization, didactic knowledge and a literature research to compare our own experiences and add additional aspects.

**Results**: Tips include the process of developing the logbook itself, the change-management process, conditions of training and the integration of logbooks into the curriculum.

**Conclusions**: Logbooks can be a valuable tool for training in clinical settings, especially when multiple sites are involved, when you take our tips into consideration.

## Introduction

### Background

To develop clinical expertise it is essential that learners receive integrated experiences, see polymorphic cases of typical diseases and can repeatedly practice their competencies in clinical settings (Regehr & Norman 1996). However, the quality of clinical teaching depends on the number and type of patients (Dolmans et al. 1999) as well as the educational expertise of the clinical teacher (Jolly 1994). Therefore, there is a great variation in trainees’ experience (Remmen et al. 2000). To ensure consistent quality and educational standards, logbooks have been introduced in clinical training. Logbooks are a collection of learning objectives and additional information concerning a specific educational period.

**Table:** 

**Practice points**
Logbooks provide a collection of learning objectives to ensure consistent educational standards in clinical training. Logbooks give a fast overview over requirements of clinical training and the status quo of fulfillment. Logbooks are especially useful if multiple sites are involved in the clinical training. In practice the use of logbooks is often deficient. To successfully implement logbooks into clinical training it is necessary to develop the logbook carefully, involve and inform stakeholders, provide the necessary resources and integrate the logbook within the curriculum.

Logbooks are used all over the world from undergraduate to postgraduate training, in human, veterinary and dental medicine, nursing schools and pharmacy, either in paper or electronic format (cf. e.g. Luke et al. 1991; Dolmans et al. 1999; Dennick 2000; Patil & Lee 2002; Dahllof et al. 2004; Merry et al. 2006; Watters et al. 2006; Chou et al. 2009; Hogg et al. 2011; Yu et al. 2011; ÄAppO 2012; Khorashadizadeh & Alavinia 2012; Nikendei et al. 2012; Witt et al. 2012; Dale et al. 2013; Jenkins et al. 2013; Nizarali et al. 2014). In Germany, national discipline related logbooks such as in surgery and internal medicine (Medizinischer Fakultätentag 2012) or in general medicine (DEGAM 2013) were developed to guarantee a standardized minimum of clinical training over all faculties/hospitals in one discipline, especially since medical students in the practical (final) year can choose their sites from all over Germany (ÄAppO 2012). Therefore, using logbooks in clinical training is a statutory requirement in the practical (final) year of medical education of the German Medical Licensure Act (ÄAppO 2012).

Logbooks provide a clear setting of learning objectives and give trainees and clinical teachers a quick overview of the requirements of training and an idea of the learning progress. Logbooks are especially useful if different sites are involved in the training to set a (minimum) standard of training. Logbooks assist supervisors and trainees to see at one glance which learning objectives have not yet been accomplished and to set a learning plan.

Logbooks facilitate communication between the trainee and clinical teacher (Patil & Lee 2002; Nikendei et al. 2012). They help to structure and standardize learning in clinical settings (Kraus et al. 2007; Busemann et al. 2012; Wolfgarten et al. 2012), especially when multiple sites are involved (Luke et al. 1991; Hunter et al. 2004; Fullhase et al. 2008; Yu et al. 2011; Dale et al. 2013). Standardization of logbooks in clinical training can increase the number of performed procedures (Helenius et al. 2002). The analysis of logbooks can reveal weak points of training (Ferrell 1991; Chu et al. 2008) and can evaluate whether trainees have fulfilled the minimum requirements of training (Tschudi et al. 2003).

In practice, however, the use of logbooks is often deficient (Kadmon et al. 2009). Some studies have shown that logbooks do not improve clinical training (Busemann et al. 2012) and are not used for learning (Dolmans et al. 1999). Sometimes clinical staff members are not aware of the existence of the logbook (Remmen et al. 1998). In their study about the didactic quality of clerkships Remmen et al. (2000) showed that only a minority of clinical teachers knew the content of the list of skills included in the logbook and only a minority of trainees used the logbook. Logbooks may be used inconsistently (Witt et al. 2012). Documentations do not always show achieved objectives and gaps (Jolly 1999). Trainees often evaluate logbooks as boring and repetitive (Shumway & Harden 2003), as bureaucratic (Busemann et al. 2012) or not well accepted (Remmen et al. 1998). Documentation can be faked (Khorashadizadeh & Alavinia 2012) by just collecting signatures without performing the learning objectives (Kraus et al. 2007). Experience and studies show that logbooks are not always completed (Dolmans et al. 1999; Jolly 1999; Kadmon et al. 2009; Wolfgarten et al. 2012). This may be due to a discrepancy between the learning outcomes in the logbook and experiences offered in the clinical setting (Raghoebar-Krieger et al. 2001a; Kraus et al. 2007) or that there are no consequences when they are not used (Denton et al. 2006).

In the summer of 2014 a workshop titled ‘Implementation of logbooks in clinical training’ was conducted with eleven participants from Germany and Switzerland involved in education during the final year and from five medical faculties to exchange experiences and best practice examples. After the workshop we searched the literature to compare the results of the workshop with findings from international experience, studies and reviews and to add missing aspects. The results of our work are summarized in this article.

Below we provide 12 tips on how to make the best use of logbooks within clinical training. The checklist in Table 1 summarizes the 12 tips.

**Table 1.** Checklist for successfully implementing logbooks into clinical training.

**Tip 1: Use all resources you can obtain and do not repeat work that has already been done**	
– Does a logbook for your discipline and stage of education already exist?	□
– Does it fulfill criteria of the tips 3–5?	□
**Tip 2: Involve all stakeholders and embed the introduction of logbooks into a change management process**	
– Are all stakeholders involved in the process of introducing the logbook and further development (supervising physicians, mentors, students)?	□
– How do you ensure transparency of the whole process?	□
**Tip 3: Keep it short, simple, and precise**	
– Are all objectives listed in the logbook really important?	□
– Are the basic skills and learning objectives exactly defined?	□
– Is additional information included (such as frequently needed knowledge of the discipline or contact details of supervising physicians and mentors)?	□
– Does the arrangement of data allow timely and easy analysis?	□
**Tip 4: Mind legal issues**	
– Do you take copyright/ownership of your country into account?	□
– Do you take data security of patients into account?	□
– Do you take data security of trainee into account?	□
**Tip 5: Use a handy logbook format**	
– If you use paper-based logbooks: is it pocket-size?	□
– If you use electronic logbooks: do you have an appropriate IT?	□
– Is the logbook of low cost?	□
**Tip 6: Make the logbook an integral part of the curriculum**	
– Are the learning objectives of the logbook part of the curriculum?	□
– Are the learning objectives of the logbook part of the curriculum in lectures and seminars (Miller-Level 1 and 2)?	
– Are the basic skills and learning objectives of the logbook part of assessment?	□
**Tip 7: Mentor and supervise learning objectives**	
– Is there regular communication and supervision via logbook between doctors and students?	□
– If in addition to the supervising physician, there is a mentor involved in the training process: is the logbook used to evaluate the learning process?	□
**Tip 8: Provide time and space for teaching and learning**	
– Do supervising physicians and mentors have enough time to supervise and to mentor?	□
– Do trainees have enough time to read, to study and to work with the logbook?	□
– Does the head of the department accept and support the logbook?	□
**Tip 9: Establish an easy going workflow**	
– Did you contact involved staff to find the best way to distribute, collect and evaluate the logbook?	□
– Does your workflow involve following activities around the logbook: printing, storing, handing over, explaining, collecting, reviewing and updating?	□
**Tip 10: Implement an evaluation cycle to optimize logbook-location-fit**	
– Is the evaluation used to improve the curriculum of the clinical setting?	□
– Do you give timely feedback to the students?	□
– Do you give timely feedback to faculty?	□
– Do you give timely feedback to the supervising physicians?	□
– Does the evaluation show the contribution of supervising physicians and mentors to the learning of the trainees?	□
**Tip 11: Inform staff and trainees**	
– Did you inform trainees about function and content of the logbook (best face to face)?	□
– Did you inform concerned staff about function and content of the logbook (e.g. physicians, head of department, nurses)?	□
– Do you plan regular information session for concerned staff?	□
**Tip 12: Train supervising physicians and mentors**	
– Do you inform about the structure, content and aim of the logbook?	
– Do you provide regular, short training of supervising physicians and mentors?	□

## Tip 1

### Use all resources you can obtain and do not repeat work that has already been done

Before you start creating a new logbook see if there is already a logbook available for the discipline and stage of education. Then involve the people in the relevant discipline to help. Let them list the minimum standards of their department.

When you are going to develop a logbook for postgraduate training you may ask the concerned medical society if a logbook or minimum standard already exists. Take all the information into account when you write the learning outcomes. Show the draft to the staff members who are involved in clinical training and let them revise it.

## Tip 2

### Involve all stakeholders and embed the introduction of logbooks into a change management process

Acceptance by staff and supervising physicians is essential in order to implement logbooks in training within clinical settings (Kadmon et al. 2009). At our workshop, participants reported best practice when several stakeholders were involved in the development and implementation of logbooks. An internal survey at the medical faculty of Tuebingen showed that supervising physicians, mentors and students wanted to be involved. Involving supervising physicians and mentors to determine the logbook contents ensures local acceptability and feasibility with the additional benefit of combining training and agreed standards. Trainees can help to identify essential additional information that is needed in the logbook. All involved persons should know that all needs are being taken into account; thus, transparency of the whole process is very important (Schmidt & Hahn 2009). Set a realistic time frame with milestones.

## Tip 3

### Keep it short, simple and precise

Logbook content should be to the point (Watters et al. 2006; Wolfgarten et al. 2012) and presented in a clear structure (Vanek et al. 1993).

Ensure that the learning objectives are achievable during the assignment (Denton et al. 2006). Fewer objectives are better (Watters et al. 2006; Busemann et al. 2012), thus list the basic skills of your subject. Denton et al. (2006) found problems with misunderstandings and completion of pre-structured logbooks with given categories. In our workshop we agreed that exactly defined learning objectives are important. Luke et al. (1991) recommend assuring the flexibility of training with core learning objectives and practical activities. Khorashadizadeh & Alavinia (2012) concluded from their interviews that logbooks should allow the fast collection of valid, relevant and reliable data. The content of the logbook has to allow timely and easy data analysis (Denton et al. 2006). In the internal survey in Tuebingen (Germany) students liked the therapy guidelines and wanted additional information about important clinical pictures. In Heidelberg students liked the organizational part of the logbook (Kraus et al. 2007). These aspects can be added to the logbook to complement the list of learning objectives. A logbook is a compromise between simplicity and comprehensiveness. With the advent of electronic logbooks this might not be such a problem in future as there can be a simple ‘front end’ with opportunities to access further information ‘in the cloud’.

## Tip 4

### Mind legal issues

Logbooks are individual learning guides for trainees. Before implementing logbooks in clinical training, be mindful of issues of copyright/ownership in your country.

Including patient data in logbooks can decrease the effort in documenting, but at the same time leads to problems of data security (Merry et al. 2006). Watters et al. (2006) recommend keeping minimal and de-identified patient data in logbooks. The same applies to the personal data of the trainee.

## Tip 5

### Use a handy logbook format

Logbooks should be convenient to carry around. Therefore, weight and size are an issue, likewise the decision between digital and print options. Watters et al. (2006) recommend from their experience adaptable digital and mobile versions of logbooks. Furthermore, electronic logbooks simplify recording and analyzing data (Merry et al. 2006; Gomez Dias et al. 2015) and allow more efficient data access (Aphinives 2013). All trainees must have access to the digital logbook but not every trainee owns his or her own mobile device. Paper-based logbooks can be filled out easily, but are difficult to analyze and archive (Denton et al. 2006). Paper-based logbooks should be pocket-size (Dent & Davis 1995; Kadmon et al. 2009) and firm (Kraus et al. 2007). Irrespective of whether they are paper or electronic logbooks should be of low cost (Denton et al. 2006; Khorashadizadeh & Alavinia 2012).

## Tip 6

### Make the logbook an integral part of the curriculum

Learning outcomes in the logbook, learning practical procedures in clinical settings and assessment at the end of the educational period should be constructively aligned in the curriculum (Biggs 1996,1999; Treleaven & Voola 2008). That means learning outcomes defined in the curriculum and the learning objectives in the logbook should correspond. If logbooks are embedded in the curriculum, they can give trainees a structure, and help them to take responsibility for their own learning process (Dennick 2000). In our workshop participants reported a better quality of clinical training when the logbook was an integral part of the curriculum, e.g. objectives of the logbook were dealt within central teaching units of the faculty. Aligning learning objectives to requirements in the following assessment increases the importance of the logbook and therefore the utility for the trainee.

## Tip 7

### Mentor and supervise learning objectives

Documentation in logbooks is not reliable when not supervised (Raghoebar-Krieger et al. 2001b). Wolfgarten et al. (2012) and participants of our workshop experienced that logbooks are often incomplete and flawed if there is no continuous mentoring. Therefore, mentoring is essential for successfully implementing training via logbooks in clinical settings (Schmidt & Hahn 2009). Schmidt & Hahn (2009) also suggest distinguishing between the role of a supervising physician and the role of a mentor: the supervising physician on the ward provides learning activities in day-to-day business and the mentor interacts with all the trainees in one department and supports their clinical development (Schmidt & Hahn 2009). Mentors and mentees can seal learning contracts to promote self-regulated learning via increasing autonomy and motivation and to foster communication (Nikendei et al. 2012; Dale et al. 2013). Supervising physicians need to review the logbook about biweekly, depending on the duration of the period, for fast information of requirements and potential learning gaps of their current trainees, whereas mentors have more time and resources to evaluate the learning progress and to use different learning tools (e.g. portfolio).

## Tip 8

### Provide time and space for teaching and learning

Experience in Germany has shown that restricted time and poor motivation of physicians has a negative impact on using logbooks in clinical training, whereas engagement of physicians in clinical training increases when they have commitment (Wolfgarten et al. 2012). Schmidt & Hahn (2009) conclude that you need resources to train with logbooks. This includes time for supervising and mentoring (Kadmon et al. 2009; Schmidt & Hahn 2009; Busemann et al. 2012) as well as time for clinical activities for trainees with less routine (Busemann et al. 2012). This emphasizes that there is a need for acceptance and appropriate support from the head of the respective department and the medical staff involved in clinical teaching.

## Tip 9

### Establish an easy going workflow

Establish an easy going workflow that involves updating logbook content, print and storage of logbooks, handing over logbooks to trainees, by faculty staff or mentors, introducing and explaining the utilization of the logbook, checking and collecting them and reviewing them after the educational period. Before implementing logbooks into clinical training, all staff members involved in the administration of logbooks, such as secretaries in the dean’s office and departments, have to be contacted to find the best way to distribute, collect and evaluate the logbooks.

## Tip 10

### Implement an evaluation cycle to optimize logbook-location-fit

Busemann et al. (2012) suggest conducting a structured evaluation of the logbooks used in clinical training. Analysis of the logbooks should be used to improve the curriculum (Dolmans et al. 1999) and to give timely feedback to trainees, faculty and supervisors (Dolmans et al. 1999). You should evaluate the contribution of supervising physicians and mentors to the learning process and evaluate the grade and quality of supervision (Watters et al. 2006).

## Tip 11

### Inform staff and trainees

Before starting, inform trainees (Dennick 2000), for example in central information sessions prior to the final year, at the beginning of the clerkships or at each rotation. Information should contain a formal introduction to logbooks and their content (Dent & Davis 1995; Remmen et al. 2000), the function of logbooks (Dennick 2000) and the recommendation to use every situation as an opportunity for learning (Jolly 1999). 

By informing the head of department, the chance that logbook training will be fully integrated in the daily routine, increases. Then explain logbooks and their purpose in team meetings to inform concerned staff, e.g. physicians and nurses. Inform supervising physicians and mentors when trainees start their first placement in clinical settings: trainees don’t yet know how to learn in clinical settings.

## Tip 12

### Train supervising physicians and mentors

Best practice examples from our workshop as well as published experiences (Schmidt & Hahn 2009; Busemann et al. 2012) see frequent training of all stakeholders using logbooks in clinical training as essential. Schmidt et al. (2010) showed that trained mentors give a more intense support to students without needing more time. Best practice reported in our workshop was to provide short, precise, frequently repeated training to supervising physicians and mentors on how to use logbooks in clinical training and to provide tips for teaching on the run, e.g. videos, trainings, brochures as well as information about structure, content and aim of the logbook. The training should also be a place for exchanging personal experiences with the logbook training.

## Conclusion

In contrast to portfolios, which focus on students’ documentation and self-reflection of their learning activities, logbooks set clear learning objectives and help to structure the learning process in clinical settings and to ease communication between trainee and clinical teacher. To implement logbooks in clinical training successfully, logbooks have to be an integrated part of the curriculum and the daily routine on the ward. Continuous measures of quality management are necessary. Then logbooks are a valuable tool for training in clinical settings, especially when multiple sites are involved.
